# Хирургическое лечение феохромоцитом

**DOI:** 10.14341/probl13283

**Published:** 2023-11-11

**Authors:** Ш. Ш. Шихмагомедов, Д. В. Реброва, Л. М. Краснов, Е. А. Фёдоров, И. К. Чинчук, Р. А. Черников, В. Ф. Русаков, И. В. Слепцов, Е. А. Згода

**Affiliations:** Санкт-Петербургский государственный университет, Клиника высоких медицинских технологий им. Н.И. Пирогова; Санкт-Петербургский государственный университет, Клиника высоких медицинских технологий им. Н.И. Пирогова; Санкт-Петербургский государственный университет, Клиника высоких медицинских технологий им. Н.И. Пирогова; Санкт-Петербургский государственный университет, Клиника высоких медицинских технологий им. Н.И. Пирогова; Санкт-Петербургский государственный университет, Клиника высоких медицинских технологий им. Н.И. Пирогова; Санкт-Петербургский государственный университет, Клиника высоких медицинских технологий им. Н.И. Пирогова; Санкт-Петербургский государственный университет, Клиника высоких медицинских технологий им. Н.И. Пирогова; Санкт-Петербургский государственный университет, Клиника высоких медицинских технологий им. Н.И. Пирогова; Санкт-Петербургский государственный университет, Клиника высоких медицинских технологий им. Н.И. Пирогова

**Keywords:** феохромоцитома, альфа-адреноблокатор, адреналэктомия, центральная вена надпочечника, резекция надпочечника

## Abstract

Данная обзорная статья содержит в кратком изложении современные представления об аспектах предоперационной подготовки, хирургического лечения и последующего наблюдения больных с надпочечниковыми феохромоцитомами. Главной составляющей медикаментозной предоперационной подготовки является применение альфа-адреноблокаторов. Необходимость их назначения всем пациентам все чаще оспаривается, особенно для пациентов без выраженной артериальной гипертензии, и все большее число публикаций демонстрирует положительные результаты хирургического лечения без применения альфа-блокаторов, выступая за индивидуальный подход и их назначение только при наличии определенных показаний. В хирургическом лечении широкое распространение получили малоинвазивные эндоскопические методы адреналэктомий: лапароскопический и ретроперитонеоскопический, в том числе с использованием их однопортовых модификаций. Ключевой в прошлом аспект операции при феохромоцитомах — максимально раннее пересечение центральной вены — с развитием хирургической техники и анестезиологического пособия перестал быть обязательным правилом для успешной адреналэктомии. Несмотря на значимое влияние пересечения данного сосуда на интраоперационную гемодинамику, хирургическая тактика с более поздним его пересечением имеет свои обоснования и не приводит к ухудшению результатов лечения. Стандартным объемом оперативного вмешательства при феохромоцитомах является адреналэктомия, однако при наличии наследственных синдромов, таких как синдром множественной эндокринной неоплазии 2 типа, нейрофиброматоз 1 типа, синдром фон Гиппеля–Линдау, возможно проведение резекции надпочечников во избежание развития хронической надпочечниковой недостаточности.

## Abstract (en)

This review article contains a summary of modern aspects of preoperative preparation, surgical treatment, and follow-up of patients with adrenal pheochromocytomas. The main component of preoperative preparation is the use of alpha-blockers. The need to prescribe them to all patients is increasingly disputed, especially for patients without severe hypertension. An increasing number of publications demonstrate positive results of treatment without the use of alpha-blockers, advocating an individual approach and the use of the drug according to certain indications. Minimally invasive endoscopic techniques of adrenalectomy have become widespread in surgical treatment. They are represented by laparoscopic and retroperitonescopic technic, including using their single-port modifications. The earliest possible intersection of the central vein in the past was considered the most important aspect of adrenalectomy for pheochromocytoma, currently, due to the development of surgical techniques and anesthesiological manuals, this has ceased to be a mandatory rule of successful surgery. Despite the significant influence of the intersection of this vessel on intraoperative hemodynamics, surgical tactics with its later intersection have their own justifications and do not lead to a deterioration in treatment results. The standard volume of surgical intervention for pheochromocytomas is total adrenalectomy, however, in the presence of hereditary syndromes, such as multiple endocrine neoplasia type 2 syndrome, neurofibomatosis type 1, von Hippel-Lindau syndrome, it is possible to perform cortical-sparing adrenalectomy.

Феохромоцитома/параганглиома (ФХЦ/ПГ) является редкой нейроэндокринной опухолью из хромаффинной ткани, способной к гиперпродукции катехоламинов. Основным этапом лечения ФХЦ/ПГ является их хирургическое удаление. Частота развития ФХЦ/ПГ относительно невелика — примерно 1 на 100 000 человек в год, среди пациентов с артериальной гипертензией их распространенность составляет 0,2–0,6% [1], что обуславливает малую информированность врачей о методах лечения данной патологии за пределами специализированных центров. Низкая распространенность ФХЦ/ПГ затрудняет выполнение крупных исследований, в связи с чем большинство публикаций, посвященных этой проблематике, основано на малых выборках клинических наблюдений, данные их, а также мнения авторов разнятся. ФХЦ является частным случаем параганглиомы надпочечниковой локализации. Основным видом лечения ФХЦ является адреналэктомия с опухолью. Данная обзорная статья содержит обобщение современных данных, касающихся предоперационной подготовки и хирургического лечения ФХЦ.

## ПРЕДОПЕРАЦИОННАЯ ПОДГОТОВКА БОЛЬНЫХ С ФХЦ

Хирургическое удаление ФХЦ ассоциировано с такими периоперационными осложнениями, как развитие гипертонического криза, тахиаритмии и неуправляемой гемодинамики [1]. На интра- и послеоперационный прогноз влияет функциональное состояние органов-мишеней, страдающих от катехоламиновой интоксикации, прежде всего это сердце, почки, головной мозг, в связи с чем одной из задач предоперационной подготовки является увеличение их функциональных резервов [2]. В качестве предоперационной подготовки всем пациентам с ФХЦ/ПГ рекомендовано применение α1-адреноблокаторов с целью улучшения интраоперационного контроля гемодинамики [3][4]. Несмотря на патофизиологическую обоснованность и ожидаемую клиническую эффективность данной терапии, у ряда авторов вызывает сомнения необходимость ее применения для всех больных с ФХЦ [5–7]. Существует ряд исследований, в которых описаны результаты успешного лечения больных с ФХЦ без применения предоперационной подготовки альфа-1 адреноблокаторами [8–12]. В исследовании Groeben H. и соавт. (2017) крупной когорты пациентов с ФХЦ/ПГ при сравнении интраоперационной гемодинамики и послеоперационных осложнений группы из 110 пациентов с предварительной альфа-блокадой и 166 больных без нее достоверных различий установлено не было [8]. При этом Castinetti F. и соавт. отмечают наличие дополнительных негативных последствий предоперационной терапии α-адреноблокаторами, помимо стандартных побочных эффектов, в виде развития послеоперационной гипотензии и, как следствие, необходимости более длительной вазопрессорной терапии [13]. Так, в недавнем крупном ретроспективном многоцентровом когортном исследовании Groeben H. и соавт. (2020) было показано даже более высокое число специфических осложнений, связанных с нестабильной гемодинамикой, в группе пациентов, получавших α-блокаторы, по сравнению с группой больных, не проходивших данной предоперационной подготовки [1]. В связи с этим рядом авторов было выдвинуто предположение о возможном ранжировании больных по степени риска с целью определения необходимости терапии альфа-адреноблокаторами [8][13][14–16]. Castinetti F. и соавт. (2022) предлагают свои методы отбора пациентов, нуждающихся в данной подготовке, в которых играют роль клиническая картина, степень повышения уровня метанефринов и другие факторы [13]. Тем не менее ввиду отсутствия крупных рандомизированных исследований, подтверждающих эффективность и безопасность хирургического удаления катехоламин-продуцирующих опухолей без предварительной фармакологической подготовки, стандартной тактикой, закрепленной в клинических рекомендациях, остается рутинное назначение α1-адреноблокаторов всем пациентам с ФХЦ/ПГ [3][4]. В предоперационной подготовке могут также использоваться β-адреноблокаторы для контроля частоты сердечных сокращений, однако важно помнить, что их использование возможно только после предварительного применения α1-адреноблокаторов по крайней мере в течение 3 сут [4][14][17].

## ВЫБОР ХИРУРГИЧЕСКОГО ДОСТУПА К НАДПОЧЕЧНИКУ

Впервые методика открытой адреналэктомии была выполнена в 1926 г. независимо друг от друга швейцарским хирургом Цезарем Ру и американским хирургом Чарлзом Мейо [18]. Несмотря на то что прошло почти 100 лет со дня первого проведения подобной операции, методика применяется до сих пор, однако в настоящее время к ней прибегают лишь при невозможности выполнить операцию малоинвазивными техниками, что обычно связано с крупными размерами опухоли, спаянностью с окружающими органами, инвазией в соседние органы и ткани или иными причинами. Наибольшим размером феохромоцитомы для использования эндоскопических методик чаще всего называли 5–6 см, однако на данный момент имеется множество публикаций, подтверждающих безопасность и эффективность эндоскопических методик в удалении опухолей и большего размера [19–21]. Одним из значимых факторов является личный опыт хирурга и операционной бригады в целом. Ранее существовавшее мнение о большей безопасности открытых адреналэктомий перед эндоскопическими в плане контроля интраоперационой гемодинамики при ФХЦ/ПГ было опровергнуто рядом исследований [22][23]. Множество публикаций посвящено сравнению двух вариантов эндоскопических адреналэктомий: лапароскопической и ретроперитонеоскопической. В ряде публикаций авторы приходят к выводу об отсутствии значимых различий в результатах лечения больных [24][25]. Другие отдают предпочтение ретроперитонеоскопической методике, в том числе при ФХЦ [26][27]. В крупном современном метаанализе Gavriilidis P. et al. (2020), включающем 775 пациентов, основными преимуществами ретроперитонеоскопической методики явилось уменьшение интраоперационной кровопотери, потребности в анальгетиках и продолжительности госпитализации [28]. Среди недостатков ретроперитонеоскопического доступа по отношению к лапароскопическому следует отметить больший риск интраоперационной гипотензии [29] и более длительный период обучения [9]. В специализированных центрах эндокринной хирургии требуется от 24 до 42 операций для полного прохождения всего курса хирургического обучения [30]. Ретроперитонеоскопический метод особенно ценен при лечении пациентов с предшествующими операциями на брюшной полости и наличием в ней спаечного процесса, в связи с чем рекомендуется включение данной техники в арсенал эндокринного хирурга [31]. Как для ретроперитонеоскопического, так и для лапароскопического доступов существуют также не столь распространенные, но тем не менее эффективно применяемые однопортовые модификации, которые обеспечивают меньший болевой синдром, лучший косметический эффект и снижают длительность госпитализации [32–34]. Данные методики применяются в технически более простых случаях, при меньших размерах опухолей надпочечников и требуют больших оперативных навыков от хирурга [35].

## ИНТРАОПЕРАЦИОННАЯ ТАКТИКА ПО ОТНОШЕНИЮ К ЦЕНТРАЛЬНОЙ ВЕНЕ НАДПОЧЕЧНИКА

Пересечение центральной вены является одним из важных этапов выполнения адреналэктомии при новообразованиях надпочечников, однако именно при хромаффинных опухолях он становится ключевым. При прекращении оттока крови по центральной вене надпочечника при ФХЦ происходит резкое снижение поступления катехоламинов из опухоли в системный кровоток, что оказывает значимое влияние на гемодинамику, управление которой требует опыта, высокой квалификации и мастерства анестестезиологической бригады. Хирургическая техника на начальных этапах развития заключалась в максимально раннем пересечении центральной вены [36]. Подобная тактика приносила хорошие результаты и была удобна для лапаротомного или лапароскопического доступов, при использовании которых искомая вена находится достаточно поверхностно. При использовании забрюшинного доступа центральная вена располагается за надпочечником, в связи с чем для ее пересечения часто требуется предварительная частичная мобилизация надпочечника, особенно нижнего полюса с подходящими к нему в данной области сосудами [37]. Walz M.K., являющийся одним из основоположников ретроперитонеоскопического доступа, утверждает, что более позднее пересечение центральной вены при данном доступе не сказывается негативно на интраоперационной гемодинамике у больного, прошедшего предоперационную подготовку α-адреноблокаторами. В описанном им протоколе операции максимально раннее пересечение надпочечниковой вены не является обязательным условием успешного исхода хирургического лечения. По Walz M.K., центральная вена пересекается на одном из первых этапов адреналэктомии в случае левосторонних операций, тогда как при правосторонних — лишь после выделения нижнего полюса надпочечника и нижней полой вены [38]. Более того, в последние годы появились публикации, в которых ряд авторов, выполняя адреналэктомии, прибегают к пересечению центральной вены одним из завершающих этапов выделения надпочечника [12][39–41]. Данная тактика оправдана тем, что, во-первых, центральная вена — не единственный сосуд, обеспечивающий венозный отток от опухоли, и после ее пересечения выделение катехоламинов в кровь полностью не блокируется, а во-вторых, перевязка главной дренажной вены приводит к усиленному кровенаполнению надпочечника, увеличивая риски кровотечения из него [12][40]. На настоящий момент нет крупных рандомизированных исследований по сравнению влияния на интраоперационную гемодинамику при адреналэктомии при ФХЦ различных тактик пересечения центральной вены надпочечника, что не позволяет сделать однозначного вывода о предпочтительности какой-либо из них.

## ОРГАНОСОХРАНЯЮЩИЕ ОПЕРАЦИИ НА НАДПОЧЕЧНИКАХ

Стандартным объемом оперативного вмешательства при ФХЦ является адреналэктомия с опухолью. Удаление одного надпочечника, как правило, не приводит к развитию хронической надпочечниковой недостаточности и не требует пожизненной гормональной заместительной терапии [42]. Однако в ряде случаев необходимо назначение глюкокортикоидных гормонов после односторонней адреналэктомии по поводу ФХЦ, особенно в случаях наличия одномоментной ко-секреции кортизола опухолью до операции [43]. У пациентов с двусторонними ФХЦ и наличием наследственных синдромов, связанных с их развитием, таких как синдром множественной эндокринной неоплазии (МЭН) 2 типа, нейрофиброматоз 1 типа (NF-1), синдром фон Гиппеля–Линдау (VHL), возможно проведение резекции надпочечников с целью сохранения глюкокортикоидной и минералокортикоидной секреции [3][4]. Ряд наследственных мутаций при ФХЦ ассоциирован с высоким риском метастазирования, в связи с чем выполнение органосохраняющих операций при наличии дефектов генов SDHB, SDHD, MAX, TMEM127, HRAS, CSDE1 и MAML3 не рекомендуется [44][45]. Сохранение ткани надпочечника у больных с синдромами МЭН 2 типа и VHL, а также NF-1 приводит к увеличению частоты развития рецидива, о чем должны быть предупреждены пациенты, однако в исследованиях показано, что при этом частота появления метастазов ФХЦ не возрастает и не снижается выживаемость больных [46]. При более детальном анализе получены данные, что риск ипсилатерального рецидива значительно ниже по сравнению с контралатеральным (0–14% против 43–57% соответственно) [44], в связи с чем возникла идея о билатеральной резекции надпочечников при наследственных синдромах. Castinetti F. и соавт. отдают предпочтение двусторонней одномоментной органосохраняющей операции при поражении обоих надпочечников ФХЦ в связи с возможностью сохранения собственной гормонопродукции [47]. Другие авторы предпочитают применение индивидуализированного подхода, основанного на интраоперационной оценке состояния ткани надпочечника: при наличии непораженной ткани надпочечника возможно выполнение его резекции с удалением опухоли с последующей адреналэктомией второго надпочечника. Чаще всего в подобных случаях полному удалению подлежит надпочечник с более крупной ФХЦ [44][48][49]. Потребность в оценке возможности проведения органосохраняющих операций обусловлена высоким риском развития осложнений, обусловленных надпочечниковой недостаточностью, таких как гипоадреналовый криз или ятрогенный синдром Иценко–Кушинга у стероидзависимых пациентов [50]. Исследования показали, что около трети одного надпочечника может быть достаточно для предотвращения развития хронической надпочечниковой недостаточности, что улучшит качество жизни больных в годы, проведенные без заместительной гормональной терапии, до возможного развития рецидива ФХЦ в оставшейся ткани надпочечника [48][51]. Тем не менее даже при выполнении органосохраняющей операции у больных с двусторонней ФХЦ около трети случаев сопровождается развитием зависимости от стероидных гормонов, по-видимому, связанной с недостаточным кровоснабжением сохраненного участка надпочечника, что следует учитывать при планировании и проведении данного типа хирургического лечения [46][50]. Сохранение центральной вены надпочечника не является обязательным при выполнении резекции органа [48].

## ПОСЛЕОПЕРАЦИОННОЕ НАБЛЮДЕНИЕ

В раннем послеоперационном периоде требуется обязательный контроль основных параметров гемодинамики пациента. В европейских клинических рекомендациях от 2014 г. указана необходимость непрерывного мониторинга уровня артериального давления в первые 24–48 ч после операции [3]. Необходимость данного контроля не вызывает сомнений, однако есть разные подходы к нему. Тогда как часть медицинских центров действительно выполняет рутинно 24-часовой постоянный мониторинг уровня АД больных в условиях реанимационного отделения после удаления ФХЦ, есть немало центров, в которых послеоперационное наблюдение может осуществляться и вне палат интенсивной терапии. По результатам наблюдений авторов, суточный контроль в отделении реанимации не снижает летальность этой категории пациентов, в связи с чем не является обязательным [1]. Ввиду наличия риска метастазирования и рецидивов ФХЦ после хирургического лечения международные и российские клинические рекомендации утверждают необходимость как минимум 10-летнего послеоперационного наблюдения [3][4][15]. Существующие балльные шкалы PASS и GAPP для оценки метастатического потенциала ФХЦ обладают ограниченной прогностической силой [52]. Учитывая высокую частоту наследственно-обусловленных феохромоцитом, всем пациентам показано генетическое исследование для определения дальнейшей тактики лечения [45], которая зависит от наличия и характера наследственной мутации, объема проведенного лечения, а также ряда клинических признаков, включающих пол, возраст, соматическое состояние здоровья, имеющиеся данные об осложнениях заболевания или наличии вторичных очагов. Пациенты со спорадическими ФХЦ подлежат 10-летнему наблюдению с целью своевременной диагностики возможных метастазов или рецидивов [15]. Пациентам с параганглиомами, крупными ФХЦ (более 5–6 см) и наследственными мутациями рекомендовано пожизненное наблюдение, включающее в последнем случае также активный скрининг других компонентов генетического синдрома [52].

## ЗАКЛЮЧЕНИЕ

Широкое распространение чувствительных лабораторных тестов и применение таких методов лучевой диагностики, как компьютерная томография и магнитно-резонансная томография, привели к увеличению прижизненного выявления ФХЦ, в том числе опухолей малых размеров. Данный факт вызывал острую необходимость совершенствования хирургических методов лечения катехоламин-продуцирующих опухолей, появление технологий операций с применением мини-инвазивных доступов, улучшение анестезиологического пособия. Несмотря на тот факт, что по гистологической классификации ВОЗ пересмотра 2017 г. ФХЦ/ПГ отнесены к злокачественным новообразованиям, развитие генетических методов исследования привело к обсуждению возможности проведения органосохраняющих операций у больных с выявленными наследственными мутациями. Большинство выводов и рекомендаций сделано на основании данных мультицентровых когортных исследований и метаанализов, так как редкость патологии обуславливает отсутствие крупных рандомизированных исследований в данной области.

## ДОПОЛНИТЕЛЬНАЯ ИНФОРМАЦИЯ

Источники финансирования. Работа выполнена по инициативе авторов без привлечения финансирования.

Конфликт интересов. Авторы декларируют отсутствие явных и потенциальных конфликтов интересов, связанных с содержанием настоящей статьи.

Вклад авторов. Все авторы одобрили финальную версию статьи перед публикацией, выразили согласие нести ответственность за все аспекты работы, подразумевающую надлежащее изучение и решение вопросов, связанных с точностью или добросовестностью любой части работы.
